# Real-Time fMRI Neurofeedback Modulation of Dopaminergic Midbrain Activity in Young Adults With Elevated Internet Gaming Disorder Risk: Randomized Controlled Trial

**DOI:** 10.2196/64687

**Published:** 2025-01-29

**Authors:** Anqi Gu, Cheng Lam Chan, Xiaolei Xu, Joseph P Dexter, Benjamin Becker, Zhiying Zhao

**Affiliations:** 1 Centre for Cognitive and Brain Sciences Institute of Collaborative Innovation University of Macau Macau China; 2 School of Psychology Shandong Normal University Jinan China; 3 Centre for Data Science Institute of Collaborative Innovation University of Macau Macau China; 4 Department of Computer and Information Science Faculty of Science and Technology University of Macau Macau China; 5 The State Key Laboratory of Brain and Cognitive Sciences The University of Hong Kong Hong Kong China; 6 Department of Psychology The University of Hong Kong Hong Kong China

**Keywords:** real-time functional magnetic resonance imaging neurofeedback, internet gaming disorder, craving, reward processing, ventral tegmental area

## Abstract

This study provides preliminary evidence for real-time functional magnetic resonance imaging neurofeedback (rt-fMRI NF) as a potential intervention approach for internet gaming disorder (IGD). In a preregistered, randomized, single-blind trial, young individuals with elevated IGD risk were trained to downregulate gaming addiction–related brain activity. We show that, after 2 sessions of neurofeedback training, participants successfully downregulated their brain responses to gaming cues, suggesting the therapeutic potential of rt-fMRI NF for IGD (Trial Registration: ClinicalTrials.gov NCT06063642; https://clinicaltrials.gov/study/NCT06063642).

## Introduction

Internet gaming disorder (IGD) had a global prevalence of 10.4% by 2022; it disproportionately affects younger individuals [1]. The effectiveness of traditional psychosocial and pharmacological treatments, however, remains inconclusive [2]. Real-time functional magnetic resonance imaging neurofeedback (rt-fMRI NF) training allows for self-regulation of brain activity patterns and shows promise for several psychiatric conditions [3]. This preregistered, randomized, single-blind study examined rt-fMRI NF as a potential intervention for IGD. Given the key role and potential modifiability of the ventral tegmental area (VTA), a dopaminergic midbrain region involved in reward processing functions [4,5], we validated the association between VTA response and gaming addiction severity in a small sample (N=9). In a larger study (N=20), we evaluated whether 2 neurofeedback training sessions for VTA downregulation reduced craving for internet gaming.

## Methods

### Overview

**Figure 1:**
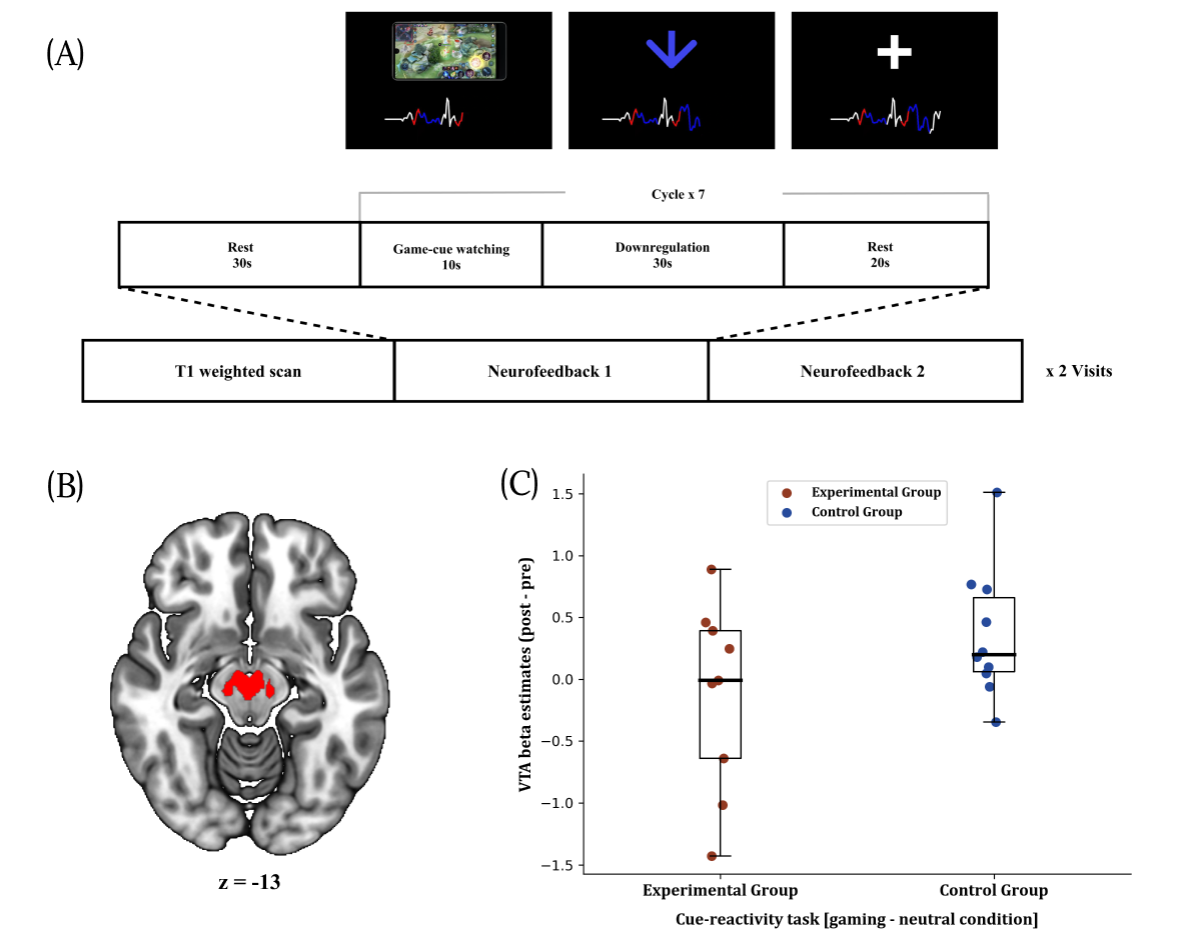
(A) The structure of the neurofeedback setting. Blood-oxygen-level–dependent activities of the trained regions for both groups are visualized as line graphs. Participants were not informed about the feedback region or its function. The blue arrow and white cross denote downregulation and rest, respectively. (B) The location of the ventral tegmental area (VTA) region of interest in the Montreal Neurological Imaging brain image is marked in red. (C) VTA cue-reactivity change (post – pre) in the cue-reactivity task based on a gaming – neutral comparison (*P*=.06).

We recruited young participants with IGD risk through social media–based advertisements. In study 1, 9 participants performed a gaming video–based cue-reactivity task (Multimedia Appendix 1, Figure S1) to establish the association between VTA cue-reactivity and IGD symptom level [5]. In study 2, 20 different participants were screened and randomly assigned to 1 of 2 groups. The inclusion criteria and screening instruments are described in Multimedia Appendix 1, Figures S2 and S3. The experimental group received feedback from the VTA (Montreal Neurological Imaging [MNI] coordinates [1, –17, –13]; 246 voxels; Figure 1B), while the control group received sham feedback from the right middle temporal gyrus, specifically an 8-mm–radius sphere centered at MNI coordinates [53, 6, –18]. This region was chosen because it is unrelated to reward processing and is not functionally connected with the VTA. Multimedia Appendix 1 describes the training details. Briefly, during neurofeedback training, blood-oxygen-level–dependent signals in the feedback regions were presented to participants as line graphs in real time (Figure 1A). Both groups were instructed to downregulate the signals using mental strategies that are helpful against craving; they reported their effort and perceived success after each run (Multimedia Appendix 1, Table S1).

Participants completed a 1-item visual analog scale (VAS) for current craving levels at baseline, after the second imaging session, and a month after the last scan; VAS scores were the primary outcome measure. The neural-level VTA cue-reactivity and inhibitory control performance in an affective go/no-go task [6] (Multimedia Appendix 1, Figure S4) measured at baseline and after the second training session were the secondary outcome measures. Analysis of the outcome measures used JASP (version 0.18.3; JASP Team) and the *nparLD* toolbox [7].

### Ethical Considerations

Both studies were approved by the Panel on Research Ethics of the University of Macau (BSERE23-APP004-ICI-M1). The neurofeedback study was preregistered at ClinicalTrials.gov (NCT06063642). Participants provided written informed consent prior to participation and were compensated with MOP 200 (US $24.96) supermarket vouchers. All data were deidentified.

## Results

In study 1, the cue-reactivity task successfully activated the VTA (*P*=.004 after correction for false-discovery rate at a whole-brain level; Multimedia Appendix 1, Figure S5A). Moreover, the VTA response was significantly correlated with addiction severity (Pearson *r*=.733; *P*=.02; Figure S5B).

Group comparison results for study 2 are shown in Table 1, and the baseline characteristics of the neurofeedback completers can be found in Multimedia Appendix 1, Table S2. Both groups showed significantly decreased subjective craving levels (*P*=.002; Multimedia Appendix 1, Figure S6) with no significant group differences (*P*=.10). Over the course of the neurofeedback sessions, the experimental group downregulated their VTA activity to a greater extent than the control group (*P*=.02). In contrast to sham feedback, VTA neurofeedback led to more pronounced decreases in VTA cue-reactivity (Figure 1C; *P*=.06), a trend that might suggest successful transfer of learned VTA regulation. No significant interaction or group effects were found for inhibitory control (accuracy and reaction time in the go/no-go task: *P*>.12 for all groups).

**Table 1:** Overall outcomes for study 2.

Outcome measures, variables, and effects				Test statistics		*P* values
**Primary (Multimedia Appendix 1, Figure S6)**						
	**Change in visual analog scale score^a^**					
		Main effect of time	*F*_1.75,__∞_=10.54		*P*<.001	
		Post hoc time effect	Pre vs post: *z*=2.41; Pre vs follow-up: *z*=3.24		Pre vs post: 2-tailed *P*_holm_=.01; Pre vs follow-up: 2-tailed *P*_holm_<.001	
**Secondary**						
	**Ventral tegmental area cue-reactivity change^b^ (Figure 1C)**					
		Group effect	Cohen *d*=–.85		1-tailed *P*=.06	
	**Go/no-go performance^c^**					
		Main effect of time (accuracy)	*F*_1,15_=8.90		*P*=.01	
		No interaction effect or group difference (reaction time and whole-brain functional magnetic resonance imaging data)	Reaction time: F_1,15_≤2.79; whole brain: not applicable		*P*>.12 (all groups)	
**Exploratory (Multimedia Appendix 1, Figure S7)**						
	**Ventral tegmental area control during neurofeedback^d^**					
		Time × group interaction	*F*_1,16_=6.98		*P*=.02	
		Post hoc main time effect	Experimental group: *t*_8_=–2.17; control group: *t*_8_=–1.63		Experimental group: 1-tailed *P*=.03; control group: 1-tailed *P*=.93	
	**Ventral tegmental area cue-reactivity change and neurofeedback control^e^**					
		Pearson correlation	*r*=.64		*P*=.004	

^a^Nonparametric time (pre vs post vs follow-up) × group (experimental vs control) ANOVA. One participant was lost to follow-up, leaving a total of 18 participants in this analysis. The changes in visual analog scale score showed a significant time effect (*P*<.001) with no group or time × group effects (*P* >.10). The post hoc paired-sample Wilcoxon signed-rank test for the time effect showed a significant pre vs post difference (pre-neurofeedback visual analog scale median: 61; post-neurofeedback visual analog scale median: 40; *z*=2.41; 2-tailed *P*_holm_=.01) and pre vs follow-up (follow-up visual analog scale median: 43; *z*=3.24; 2-tailed *P*_holm_<.001). *P*_holm_ indicates that the Bonferroni-Holm method was used to control for false positives.

^b^Time (pre vs post) × group (experimental vs control) analysis of covariance controlling for age and sex. The experimental group showed greater reduction in cue-reactivity compared to the control group (experimental group median change: –0.37; control group median change: –0.15; 1-tailed *P*=.06).

^c^Time (pre vs post) × group (experimental vs control) ANOVA (accuracy, reaction time and whole-brain functional magnetic resonance imaging data). Go/no-go task data were lost for 2 participants due to technical issues, leaving a total of 17 participants. Accuracy on the go/no-go task improved for both groups after intervention (pre-neurofeedback median: 0.67; post-neurofeedback median: 0.83; *P*=.01); no other main or interaction effects were found for behavioral performance (*P*>.12 for all groups). No brain activation results remained significant after correction for false-discovery rate at a whole-brain level.

^d^Time (day 1 vs day 2) × group (experimental vs control) ANOVA. Data were lost for 1 participant from the third run of training, resulting in 18 participants being included in this analysis. During neurofeedback training, there was a significant interaction effect of time (day 1 vs day 2) and group (real vs sham feedback) for downregulated activation (*P*=.02). A post hoc *t* test indicated that ventral tegmental area activity decreased significantly for the experimental group (median change: –0.07; *P*=.03) but not for the control group (median change: 0.11; *P*=.93).

^e^Ventral tegmental area activity change in the cue-reactivity task was positively correlated with neurofeedback training (day 2 – day 1; *r*=.64; *P*=.004).

## Discussion

We previously proposed rt-fMRI NF as a promising therapeutic strategy for individuals with internet addiction [3]. The efficacy of rt-fMRI NF, however, has not been empirically examined for any behavioral addictions. In this trial, we found that individuals with problematic internet gaming behaviors could learn to downregulate their VTA activity through genuine feedback, and that this effect translated into changes in VTA reactivity to gaming stimuli. Although we found no improvements at the behavioral level after NF, the neural activity findings underscore the potential of VTA NF training in restoring reward processing functions in individuals with IGD risk.

The study has several limitations. First, our participants were not diagnosed with IGD, meaning that their symptoms might not have been severe enough to benefit from the intervention. Furthermore, due to the small sample size, this study was potentially underpowered to detect group differences in behavioral changes. Larger-scale trials are needed to further examine the clinical efficacy of this intervention protocol.

## Appendix

Multimedia Appendix 1 Full description of study design and methods.

Multimedia Appendix 2 CONSORT-eHEALTH checklist (V 1.6.1).

## Abbreviations

IGD: internet gaming disorder
MNI: Montreal Neurological Imaging
rt-fMRI NF: real-time functional magnetic resonance imaging neurofeedback
VAS: visual analog scale
VTA: ventral tegmental area

## Data Availability

Data will be made publicly available at the time of publication at OSF Registries [8].
