# Supplementary material for: Real-Time fMRI Neurofeedback Modulation of Dopaminergic Midbrain Activity in Young Adults With Elevated Internet Gaming Disorder Risk: Randomized Controlled Trial
Source: J Med Internet Res. 2025 Jan 29;27:e64687. doi: 10.2196/64687 (PMC11822309; doi:10.2196/64687)
Supplement: Multimedia Appendix 1 [file jmir_v27i1e64687_app1.docx]

**Multimedia Appendix 1**

**Supplemental Methods**

#### Game Selection

We chose *“King of Glory”* because in the Chinese gaming market, mobile games are dominant [1]. Among all types of mobile games, *“King of Glory”*, as a Multiplayer Online Battle Arena (MOBA) game, holds a prominent position in this industry, boasting over 200 million users, with a daily active user base exceeding 100 million [2]. Despite the popularity of mobile MOBA games, previous empirical studies have pointed out that MOBA games need to be criticized for their negative gaming environment [3] and the exacerbation of Internet gaming disorder (IGD) through frustration-driven continuance [4]. Hence, we have selected "King of Glory" as our target game.

#### Participants – study 1

A total of 10 participants who self-identified as regular players of the online mobile game “*King of Glory”* were recruited in Macau through online social media advertisement. They were administered the DSM-5 self-report scale and the short version of Young’s Internet Addiction Test [5] (sIAT, with language modified for IGD). sIAT encompasses two factors, one assessing craving/social problems and another assessing loss of control/time management, with 6 items each, both of which have been associated with variations in the architecture of key regions of the reward system. Inclusion criteria for this study included self-identified as Chinese, right-handed, ability to give informed consent, endorsing ⩾ 5 items of the proposed DSM-5 criteria for IGD, scoring ⩾ 36 on sIAT, playing “*King of Glory”* ⩾ 14 h/week for ⩾1 year and playing the game for more than 3 years. Measuring gaming duration helps us confirm that participants are still actively engaged in gaming recently, facilitating a comparison of their gaming behaviors before and after training. According to a recent study focused on the Chinese youth, 14 hours was the average number of online gaming hours per week [6]. Exclusion criteria included any MRI contraindication, any history of brain injury or surgery, or currently on psychotropic medication. These participants did not take part in study 2.

#### Participants – study 2

Participants were instructed to refrain from playing the game for at least 18 hours prior to the experiment. This abstinence protocol ensured the saliency of the gaming stimuli and consequently sufficient VTA response to down-regulate by the participants [7].

**Inclusion Criteria:**

- Meet at least five of the Diagnostic and Statistical Manual of Mental Disorders fifth edition (DSM-5) criteria for IGD and score 31 or above on the Internet Addiction Test (IAT)
- First language is Chinese (Mandarin)
- Right-handed
- Play “*King of Glory”* ⩾ 14 h/week for ⩾1 year
- Have played the mobile game "King of Glory" for more than 3 years
- Ability to give informed consent
- Normal or corrected-to-normal vision

**Exclusion Criteria:**

- Any primary diagnosis of a current psychological or neurological disorder
- Any history of psychological or neurological disorder
- Any MRI contraindication
- Currently on a psychotropic medication
- Any history of substance dependence
- Any history of brain injury or surgery

Other details of the current trial can be found on: <https://classic.clinicaltrials.gov/ct2/show/NCT06063642.>

#### Study 2 Experimental Procedures

Participants in both groups completed two MRI sessions and received a total of four runs of neurofeedback training (two runs per session) before and after which they completed cue-reactivity and affective go/no-go tasks.

The cue-reactivity paradigm used 20-second video clips captured from the game “*King of Glo*ry”. These short video clips covered scenarios such as fights between the game characters controlled by players that might trigger the desire to play. They were first rated for emotional valence and arousal level with integers from 1 to 5, with 1 representing a state of extreme sadness or calmness, and 5 representing a state of extreme joy or excitement, by an independent sample of players (N = 10) who did not participate in either of the formal studies. The twenty gaming clips deemed both rewarding and provocative (mean Valence = 3.3 ± 0.97 SD; mean Arousal = 3.37 ± 0.99 SD) were included in the cue-reactivity task for evoking VTA reward-processing activity. The cue-reactivity task also included calming Italian documentary landscape videos [8] with neutral valence and low arousal as the control condition. This task was divided into 4 runs, with 6 minutes per run, including 5 gaming and 5 neutral landscape video clips.

Participants also completed one run of go/no-go task pre- and post-neurofeedback, each lasting nine minutes. Each run included 216 trials distributed across 12 blocks. In the go/no-go task, words with happy, neutral, and fearful emotions were presented on the screen. When the words were in regular font, participants were required to respond as quickly as possible. However, when the words were in italics, participants were instructed not to respond. Each word appeared for 30 milliseconds, followed by a 900-millisecond period in which participants could execute the corresponding response (either pressing a key or refraining from pressing).

Self-reports on craving levels and IGD symptoms were collected at baseline, after the second imaging session, and a month later following the last scan. At baseline and followup, participants' gaming durations over the past month (number of days they played games in a week and number of hours played per day) were collected, along with the scores on the sIAT.

#### Neurofeedback Training

Both groups underwent two training visits with 2 runs per visit. During each training run, gaming videos were presented for 10 seconds for the participants to view, followed by 30-second self-regulation blocks (7 blocks per run). The neurofeedback training used a total of 28 gaming video clips created in the same manner as for the cue-reactivity task. Participants were informed before the first neurofeedback run that their goal was to decrease brain activity as indicated by the line graph when a blue downward arrow appeared on the screen. The line graph was updated by each scan volume (repetition time = 2000ms). They received a booklet that described potentially effective strategies, including imagining negative consequences of gaming, re-evaluating thoughts about gaming, engaging in reality checks, shifting attention, and suppressing game-related thoughts [9–11]. They were encouraged to explore the most effective strategy (including but not limited to the ones provided). They were also reminded to maintain a steady and natural breathing pattern during the downregulation process. After each neurofeedback training run, participants were asked to rate their level of self-regulation effort and self-perceived success in regulating their neural activity on a scale from 1 to 10. Participants were only informed about which ROI they received neurofeedback from after completion of the study.

#### Imaging Parameters and Offline Preprocessing

For both studies, MRI data were collected on a 3-Tesla MAGNETOM Prisma Fit system (Siemens Medical Solutions, Erlangen, Germany; version VE11B) using a 64-channel head coil with identical sequences. We used the same T2* weighted echo-planar imaging sequence (with various length) for all the functional scans: GRAPPA factor = 2, bandwidth = 2520 Hz/Px, TR = 2000ms, TE = 30ms, flip angle = 80 degrees, voxel size = 3.1×3.1×3.0 mm, FoV = 200mm^2^, thirty-seven interleaved AC-PC aligned slices per volume. To assist spatial registration to the standard space, high-resolution structural data were collected with an MPRage sequence: TR = 2300ms, TE = 2.32ms, flip angle = 8 degrees, 1mm^3^ isotropic voxel, FoV = 240mm^2^, 192 sagittal slices per volume.

For offline analyses, all functional data were first preprocessed by DPABI [12] (version7.0) using the following standard procedures: removal of the first five volumes, slice timing correction, motion correction, regressing out the white matter and cerebrospinal signals, normalization to the MNI template by means of DARTEL segmentation of the T1 structural images, and spatial smoothing (6mm FWHM kernel). A functional run was discarded if the average frame-wise displacement was greater than 0.25mm during the run.

#### Generalized Linear Models (GLM)

We used SPM12 to build GLMs for the three tasks (cue-reactivity, go/no-go, neurofeedback down-regulation training) separately with all but the rest condition included in the models as task regressors. The six rigid-body head movement parameters [13] and scanner drift (first-order) were added as nuisance regressors.

Specifically, for cue-reactivity task, the beta values for the VTA brain region were extracted based on the [gaming – neutral] contrast. The beta values were averaged separately for the pre-training and post-training runs, serving as a secondary measurement for assessing control over VTA activity. In study 1, we averaged the beta values for all four runs.

For the go/no-go task, the beta values for the [nogo - go] contrast in the dlPFC were extracted and used as a secondary measurement to assess inhibitory control.

For neurofeedback task, average beta values during downregulation were extracted from the target region of the VTA region and MTG region. This downregulation value averaged across two runs in the same day (separately at day1 and day2) served as the measurement for VTA regulation capability change.

#### Statistical Analyses

Given the ordinal nature of the VAS scores, a non-parametric ANOVA-type test was used to assess the changes in VAS scores. This analysis included two factors: Time (pre-intervention, post-intervention, 1-month followup) and Group (real and sham feedback). Changes in VAS scores from pre- to post-intervention served as the primary outcome measurement.

The change in VTA cue-reactivity and DLPFC inhibitory control were secondary outcome measurements in this study. The VTA activation level during the cue-reactivity task was computed using GLM analyses and extracted as beta-estimates averaged over the voxels from the neurofeedback ROI. Subsequently, we conducted an 1-tailed independent samples t-test on the extracted VTA cue-reactivity to compare the pre- to post-neurofeedback changes between the two groups. The inhibition control changes included accuracy and reaction time in the go/no-go task, along with DLPFC activity in inhibitory condition (no-go or go). For the ANOVA of the go/no-go behavioral data, two factors were considered: Time (pre or post), Group (real or sham feedback). The whole-brain analysis for the go/no-go fMRI data was conducted using MRM in the form of repeated measures ANOVA: Time (pre or post) and Group (real or sham feedback). We also extracted the activity of the bilateral DLPFC using a thresholded synthetic mask downloaded from Neurosynth [14]. Extracted DLFPC activity change (post - pre) was compared between the groups using an independent samples t-test.

#### Online Preprocessing of Neurofeedback Signal

Online fMRI data preprocessing and neurofeedback were performed using in-house MATLAB (The MathWorks Inc.; Natick, Massachusetts) scripts based on spm12. After reconstruction by the scanner console, raw images were transferred immediately to a computer via TCP/IP connection for preprocessing with our neurofeedback modules. In each session, before the neurofeedback training started, the T1-weighted scan was segmented and normalized into MNI space using the DARTEL approach [21]. During neurofeedback, all the functional images were realigned to the first image. Then the anatomical ROIs were registered to the realigned functional images. Extracted ROI signals were despiked with a Kalman filter. BOLD signals from a rectangular ROI placed in an adjacent slice (30 × 30 × 3 mm in AC–PC orientation, center of mass = [0, −16, −5], MNI coordinates) encompassing brain white matter area were subtracted from the ROI signals to remove nonneuronal background noises. These preprocessed ROI signals were sent to the presentation computer and visualized as line graph feedback using a Python 3.6-programed interface.

**Supplemental Figures and Tables**


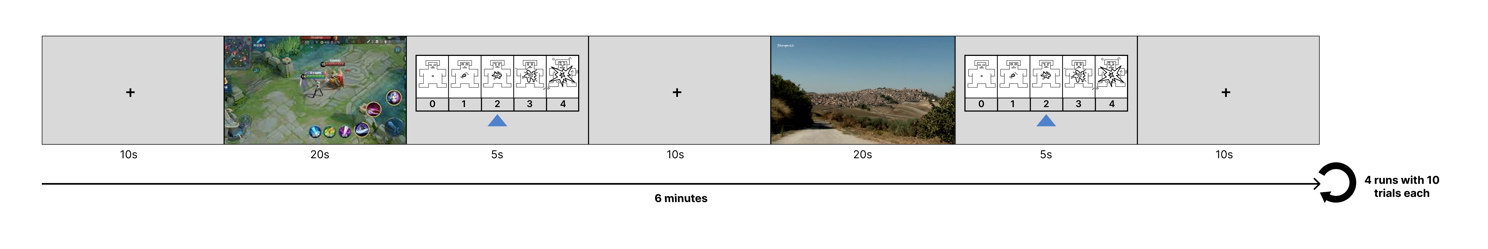


**Figure S1.** The cue-reactivity task for study 1 and study 2. Two types of video stimuli (gaming-related and neutral landscape) were provided, and the participants were asked to rate the arousal level of each video.

**
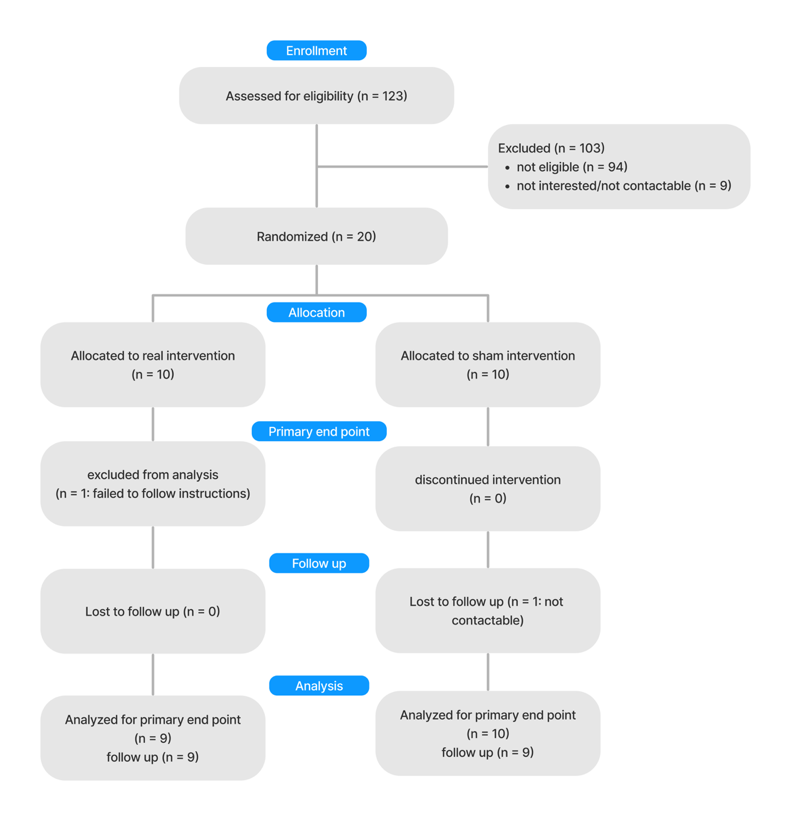
**

**Figure S2.** CONSORT flow diagram.


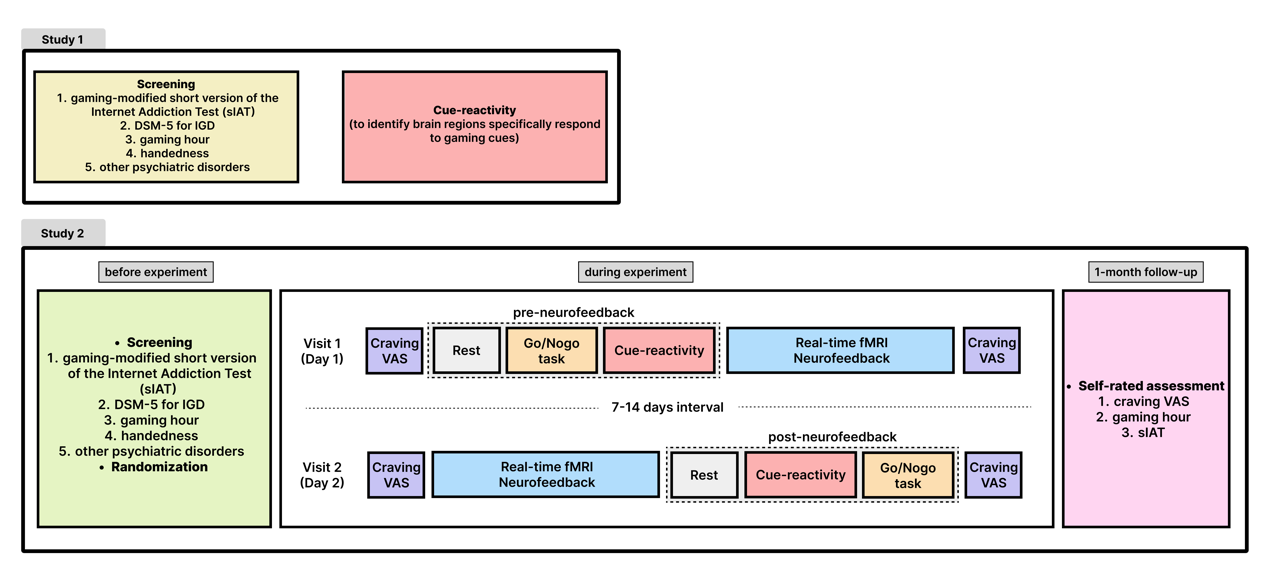
**Figure S3.** Layout of the experimental procedures

**
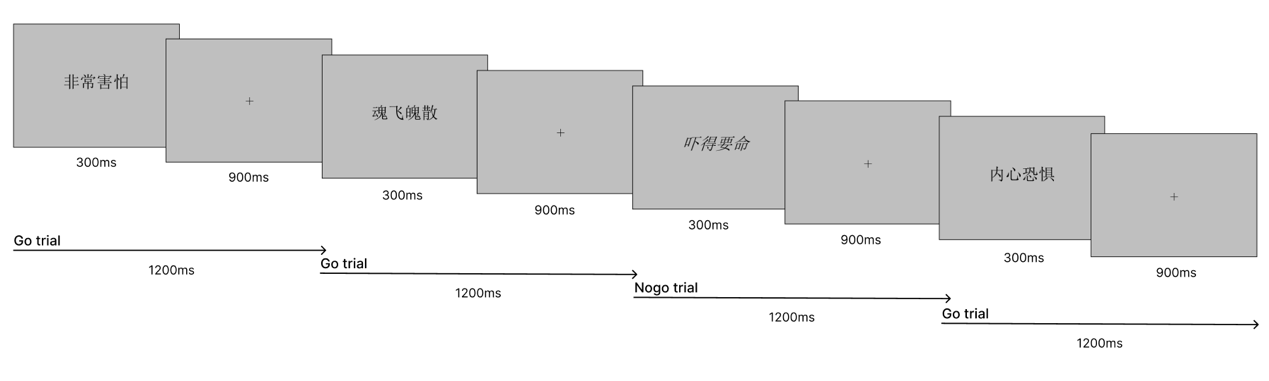
**

**Figure S4.** Layout of the affective go/no-go trials.


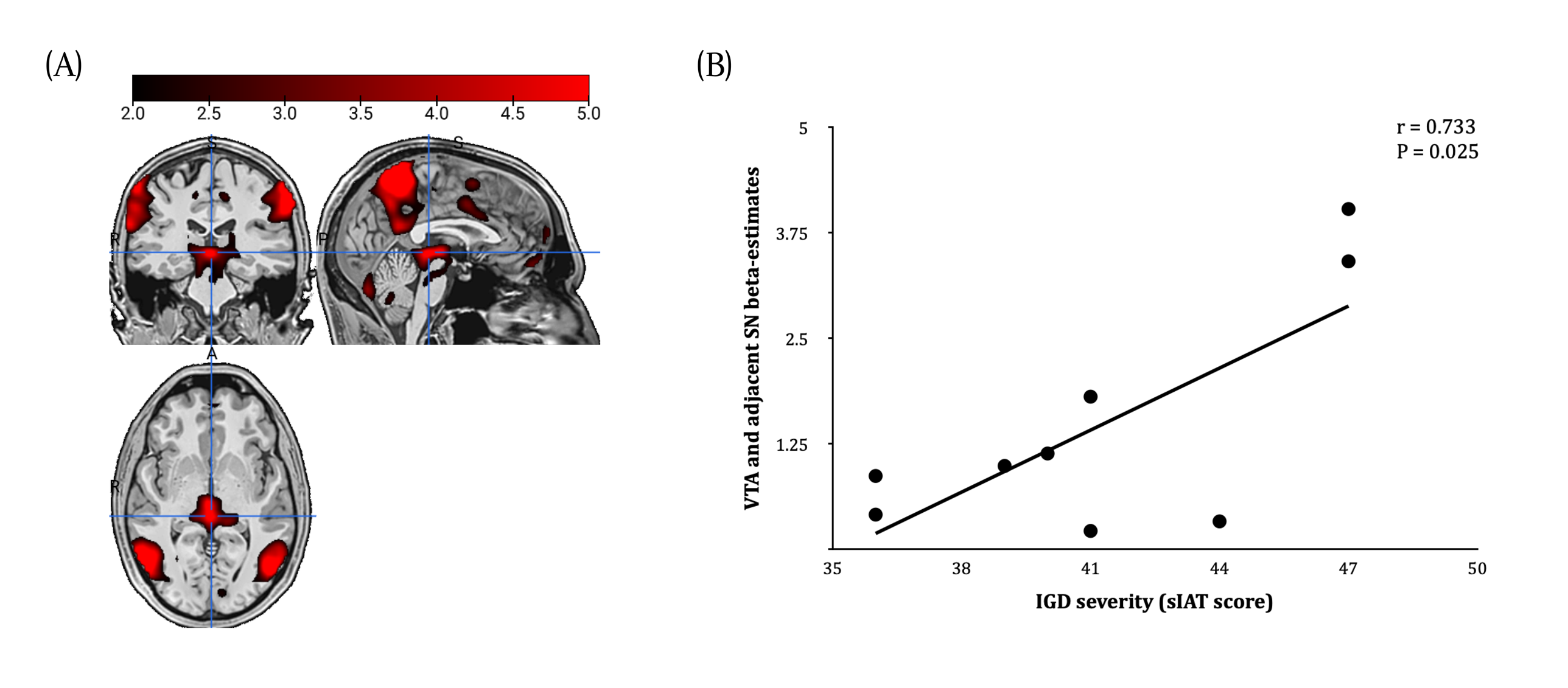


**Figure S5.** (A) Brain activation patterns in the cue-reactivity data of study 1. Individual activation data was extracted from the [Gaming – Neutral] contrast after correction (*P_FDR_* < .05). Color bar denotes t-value. (B) The positive correlation between the sIAT score and beta-value extracted from VTA and its adjacent substantia nigra (SN) area in the nine participants of study 1.

###
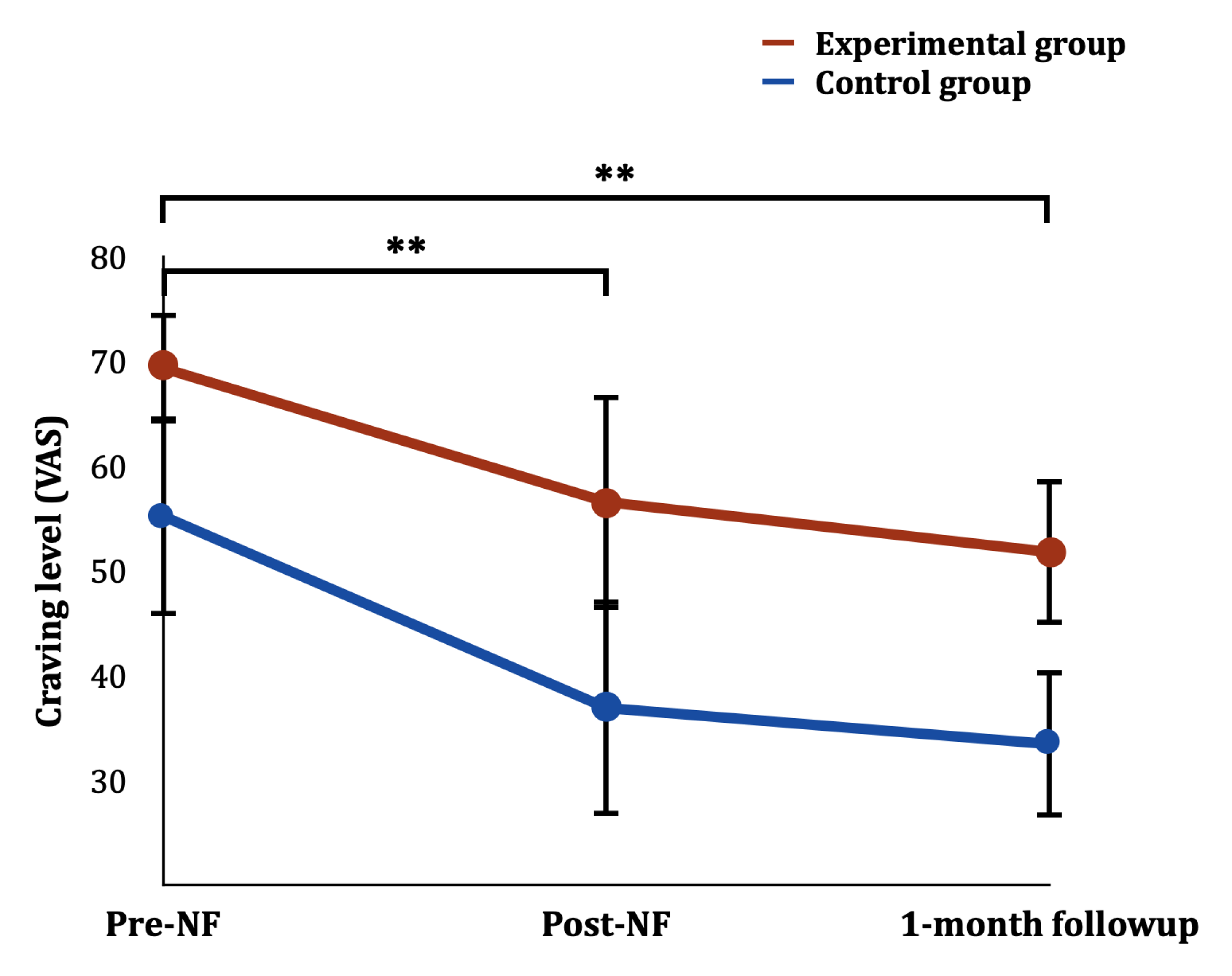


**Figure S6.** Changes in craving level over pre-training, post-training and 1-month followup in study 2. The craving VAS score decreased significantly for both groups.

Error bars denote standard errors. ** *P* ≤ .01

**Figure S7.** (A) VTA down-regulation success during neurofeedback training. VTA activation change (day 2 – day 1) in neurofeedback training based on [downregulation] contrast. (B) VTA activation in the four training runs. The VTA activation during the four neurofeedback training runs decreased significantly for experimental group. Error bars denote standard error. (C) Positive correlation between the VTA activation change in cue-reactivity task and neurofeedback training. The change of VTA activation in cue-reactivity task (post - pre) is positively correlated to the change of VTA activation in neurofeedback training (day 2 – day 1). (D) rMTG activation in the four training runs. The rMTG activation during the four neurofeedback training runs presented no significant changes for both groups. Error bars denote standard error.

**Table S1.** Reported down-regulation strategies used in neurofeedback training and self-evaluated effort and success*.* After each training run, participants were asked to report the strategies they used to down-regulate their brain activity and their perceived self-regulation effort and success during the last run of training.

|  | **Re-evaluate thoughts about gaming, times (%)** | **Reality check, times (%)** | **Imagine negative gaming effects, times (%)** | **Shift attention, times (%)** | **Inhibite game-related thoughts, times (%)** |
| --- | --- | --- | --- | --- | --- |
| **Experimental group** | 7 (10) | 19 (27) | 19 (27) | 25 (36) | 0 (0) |
| **Control group** | 12 (15) | 13 (16) | 21 (26) | 28 (35) | 6 (8) |
|  | Perceived effort | | Perceived success | | |
| **Group difference^a^** | t_17_ = 1.84, 2-tailed *P* = .08 | | t_17_ = 1.5, 2-tailed *P* = .15 | | |

^a^ Independent t-tests for two groups.

**Table S2.** Baseline characteristics of Study 2 completers

|  | **Experimental group**  **(n = 9)** | **Control group**  **(n = 10)** |  |
| --- | --- | --- | --- |
|  | **Mean (SD)** | **Mean (SD)** | ***P*** |
| **Age, Years** | 22.67 (2.35) | 21.9 (2.28) | .28 |
| **DSM-5 IGD** | 6.33 (1.23) | 6.4 (1.27) | .91 |
| **sIAT** | 39.22 (4.87) | 37.3 (3.47) | .33 |
| **BDI-II** | 10.22 (9.73) | 12.9 (9.33) | .55 |
| **STAI-state** | 36.67 (10.26) | 40.1 (10.63) | .49 |
| **STAI-trait** | 44.11 (13.02) | 49.6 (11.5) | .34 |
|  | **Count, n (%)** | **Count, n (%)** |  |
| **Male** | 6 (66.67) | 3 (30) | .11 |

**Table S3.** CRED-nf best practices checklist 2020

| Domain | Item # | Checklist item | Reported on page # |
| --- | --- | --- | --- |
| Pre-experiment | | | |
|  | 1a | Pre-register experimental protocol and planned analyses | #2 |
|  | 1b | Justify sample size | #5 |
| Control groups | | | |
|  | 2a | Employ control group(s) or control condition(s) | #7-8 |
|  | 2b | When leveraging experimental designs where a double-blind is possible, use a double-blind |  |
|  | 2c | Blind those who rate the outcomes, and when possible, the statisticians involved |  |
|  | 2d | Examine to what extent participants and experimenters remain blinded |  |
|  | 2e | In clinical efficacy studies, employ a standard-of-care intervention group as a benchmark for improvement |  |
| Control measures | | | |
|  | 3a | Collect data on psychosocial factors | #6 |
|  | 3b | Report whether participants were provided with a strategy | #7 |
|  | 3c | Report the strategies participants used | Supplementary material #5 |
|  | 3d | Report methods used for online-data processing and artefact correction |  |
|  | 3e | Report condition and group effects for artefacts |  |
| Feedback specifications | | | |
|  | 4a | Report how the online-feature extraction was defined | #7 |
|  | 4b | Report and justify the reinforcement schedule | #7 |
|  | 4c | Report the feedback modality and content | #7 |
|  | 4d | Collect and report all brain activity variable(s) and/or contrasts used for feedback, as displayed to experimental participants | #6-7 |
|  | 4e | Report the hardware and software used | #7 |
| Outcome measures | | | |
| Brain | 5a | Report neurofeedback regulation success based on the feedback signal | #12 |
|  | 5b | Plot within-session and between-session regulation blocks of feedback variable(s), as well as pre-to-post resting baselines or contrasts | #11 |
|  | 5c | Statistically compare the experimental condition/group to the control condition(s)/group(s) (not only each group to baseline measures) | #10-12 |
| Behaviour | 6a | Include measures of clinical or behavioural significance, defined a priori, and describe whether they were reached | #10 |
|  | 6b | Run correlational analyses between regulation success and behavioural outcomes | #13 |
| Data storage | | | |
|  | 7a | Upload all materials, analysis scripts, code, and raw data used for analyses, as well as final values, to an open access data repository, when feasible |  |

**Supplemental references**

1. Wang Y. Development and Prospect of China Mobile Game Market. Highlights Bus Econ Manag. 2023 Feb 16;5:355–9.

2. Sun Z. The Impact of Social Media Marketing on the Mobile Game Industry--Take Glory of the Kings as an Example. Highlights Bus Econ Manag. 2023 Dec 29;23:205–12.

3. Wang Y, Dai Y, Chen S, Wang L, Hoorn JF. Multiplayer Online Battle Arena (MOBA) Games: Improving Negative Atmosphere with Social Robots and AI Teammates. Systems. 2023 Aug 14;11(8):425.

4. T’ng ST, Ho KH, Pau K. Need Frustration, Gaming Motives, and Internet Gaming Disorder in Mobile Multiplayer Online Battle Arena (MOBA) Games: Through the Lens of Self-Determination Theory. Int J Ment Health Addict. 2023 Dec;21(6):3821–41.

5. Young KS. Caught in the net: how to recognize the signs of Internet addiction--and a winning strategy for recovery. New York: J. Wiley; 1998. 248 p.

6. Liao Z, Huang Q, Huang S, Tan L, Shao T, Fang T, et al. Prevalence of Internet Gaming Disorder and Its Association With Personality Traits and Gaming Characteristics Among Chinese Adolescent Gamers. Front Psychiatry. 2020 Nov 17;11:598585.

7. Fernandez DP, Kuss DJ, Griffiths MD. Short-term abstinence effects across potential behavioral addictions: A systematic review. Clin Psychol Rev. 2020 Mar;76:101828.

8. Maffei A, Angrilli A. E-MOVIE - Experimental MOVies for Induction of Emotions in neuroscience: An innovative film database with normative data and sex differences. Greco A, editor. PLOS ONE. 2019 Oct 3;14(10):e0223124.

9. Brühl AB, Scherpiet S, Sulzer J, Stämpfli P, Seifritz E, Herwig U. Real-time Neurofeedback Using Functional MRI Could Improve Down-Regulation of Amygdala Activity During Emotional Stimulation: A Proof-of-Concept Study. Brain Topogr. 2014 Jan;27(1):138–48.

10. Herwig U, Lutz J, Scherpiet S, Scheerer H, Kohlberg J, Opialla S, et al. Training emotion regulation through real-time fMRI neurofeedback of amygdala activity. NeuroImage. 2019 Jan;184:687–96.

11. Kirschner M, Sladky R, Haugg A, Stämpfli P, Jehli E, Hodel M, et al. Self-regulation of the dopaminergic reward circuit in cocaine users with mental imagery and neurofeedback. EBioMedicine. 2018 Nov;37:489–98.

12. Yan CG, Wang XD, Zuo XN, Zang YF. DPABI: Data Processing & Analysis for (Resting-State) Brain Imaging. Neuroinformatics. 2016 Jul;14(3):339–51.

13. Friston KJ, Williams S, Howard R, Frackowiak RSJ, Turner R. Movement‐Related effects in fMRI time‐series. Magn Reson Med. 1996 Mar;35(3):346–55.

14. neurosynth [Internet]. Available from: https://www.neurosynth.org
